# Integrative genomic and single-cell framework identifies druggable targets for colorectal cancer precision therapy

**DOI:** 10.3389/fimmu.2025.1604154

**Published:** 2025-05-27

**Authors:** Yanggang Hong, Jiajun Li, Nuo Xu, Wanyi Shu, Feng Chen, Yuze Mi, Haigang Geng, Qian Li

**Affiliations:** ^1^ State Key Laboratory of Systems Medicine for Cancer, Shanghai Cancer Institute & Department of Liver Surgery, Renji Hospital, Shanghai Jiao Tong University School of Medicine, Shanghai, China; ^2^ The Second Affiliated Hospital and Yuying Children’s Hospital of Wenzhou Medical University, Wenzhou, Zhejiang, China; ^3^ Wenzhou Medical University, Wenzhou, Zhejiang, China; ^4^ Department of Gastrointestinal Surgery, Renji Hospital, Shanghai Jiao Tong University School of Medicine, Shanghai, China; ^5^ Oncology Department of Shanghai Sixth People’s Hospital Affiliated to Shanghai Jiao Tong University School of Medicine, Shanghai, China

**Keywords:** colorectal cancer, genetics, druggable targets, single-cell transcriptomics, precision oncology

## Abstract

**Background:**

Colorectal cancer (CRC) remains a leading cause of cancer-related mortality worldwide. Despite therapeutic advances, there is a critical need to identify novel, effective, and safe drug targets to improve precision treatment strategies.

**Methods:**

We developed a multi-layered framework integrating Mendelian randomization (MR), colocalization analysis, genome-wide association study (GWAS) data, and expression quantitative trait loci (eQTLs) to prioritize causal and druggable genes in CRC. Single-cell and bulk RNA sequencing were used to characterize gene expression within the tumor microenvironment. Phenome-wide association studies (PheWAS) assessed off-target effects, and drug repurposing potential was evaluated using OpenTargets, DrugBank, and DGIdb. Validation of key targets was performed through RT-qPCR and immunohistochemistry (IHC) in CRC patient samples.

**Results:**

Out of 4,479 druggable genes, MR analysis identified 47 candidates significantly associated with CRC risk. Six genes (TFRC, TNFSF14, LAMC1, PLK1, TYMS, and TSSK6) demonstrated strong colocalization signals and were further validated across replication datasets and subtype-stratified analyses. PheWAS analysis revealed minimal off-target effects for these genes. Notably, several of these genes have already been targeted by existing or investigational drugs, suggesting potential for repurposing. These genes exhibited distinct expression patterns in tumor and stromal cell types and were differentially expressed in CRC versus normal tissues. Among them, TNFSF14, an immune modulator, is particularly involved in regulating T cell activation within the tumor microenvironment.

**Conclusion:**

This study identifies and validates six promising druggable targets for CRC, providing a strong foundation for future preclinical studies. These findings open avenues for advancing precision oncology and drug repurposing strategies in CRC treatment, contributing to the development of more effective and personalized therapeutic approaches.

## 1 Introduction

Colorectal cancer (CRC) is one of the most prevalent and lethal malignancies worldwide, with an increasing incidence in both developed and developing countries. According to the Global Cancer Statistics, CRC ranks as the third most common cancer and the second leading cause of cancer-related mortality globally (1). The pathogenesis of CRC is a complex interplay of genetic, environmental, and lifestyle factors, including diet, smoking, alcohol consumption, and chronic inflammation (2). The adenoma-carcinoma sequence describes the progression of normal colonic epithelium to adenomatous polyps and ultimately invasive carcinoma, driven by accumulated genetic and epigenetic alterations (3). Mutations in key genes such as APC, TP53, KRAS, and PIK3CA, alongside microsatellite instability (MSI) and CpG island methylator phenotype (CIMP), are crucial in CRC pathogenesis (4, 5). Despite advances in early detection through colonoscopy and fecal immunochemical tests, a significant proportion of CRC cases are diagnosed at an advanced stage, leading to poor survival outcomes (6).

The development of anti-cancer drugs has significantly transformed CRC treatment, yet challenges remain due to drug resistance, toxicity, and limited efficacy in certain patient subgroups. Conventional treatment strategies include surgical resection for localized disease, combined with chemotherapy and other neoadjuvant treatments such as targeted therapy or immunotherapy (7). The introduction of fluoropyrimidine-based chemotherapy (e.g., 5-fluorouracil) in combination with oxaliplatin or irinotecan has improved survival rates (8, 9). Targeted therapies against EGFR (cetuximab, panitumumab) and VEGF (bevacizumab) have further enhanced treatment options for metastatic CRC, particularly in patients with RAS wild-type tumors (10, 11). More recently, immune checkpoint inhibitors (ICIs) have revolutionized the treatment landscape for MSI/dMMR CRC, demonstrating durable responses and improved overall survival (12). However, resistance mechanisms, heterogeneity in drug response, and high treatment costs continue to impede the widespread success of these therapies, necessitating the exploration of novel drug targets.

Recent studies have highlighted the role of tumor-initiating cells (TICs), a subpopulation with stem-like properties and high tumorigenic potential, in contributing to immune evasion, drug resistance, and disease recurrence (13). TICs remodel the tumor microenvironment to suppress immune responses, facilitating tumor progression and therapeutic failure. Therefore, identifying immune-relevant and druggable targets within TIC-enriched populations represents a promising approach to overcome resistance and enhance the efficacy of immunotherapy in digestive system tumors such as CRC.

Several studies have also sought to identify novel therapeutic targets in CRC, leveraging insights from genomics, transcriptomics, and proteomics. For example, inhibition of BRAF V600E-mutant tumors using vemurafenib in combination with cetuximab has shown promising but limited clinical efficacy, highlighting the need for combination strategies. HER2-targeted therapies, such as trastuzumab and pertuzumab, have been explored in HER2-amplified CRC with encouraging results (14, 15). Other emerging drug targets include Wnt signaling inhibitors (Porcupine inhibitors), MEK inhibitors, and metabolic regulators such as IDH1/2 inhibitors (16). Additionally, RNA-based therapies, including small interfering RNA (siRNA) and antisense oligonucleotides, have demonstrated preclinical potential in targeting oncogenic pathways in CRC (17). Despite these advancements, many of these therapies remain in early development stages or face challenges in clinical translation due to toxicity, off-target effects, and tumor heterogeneity.

**Figure 1:**
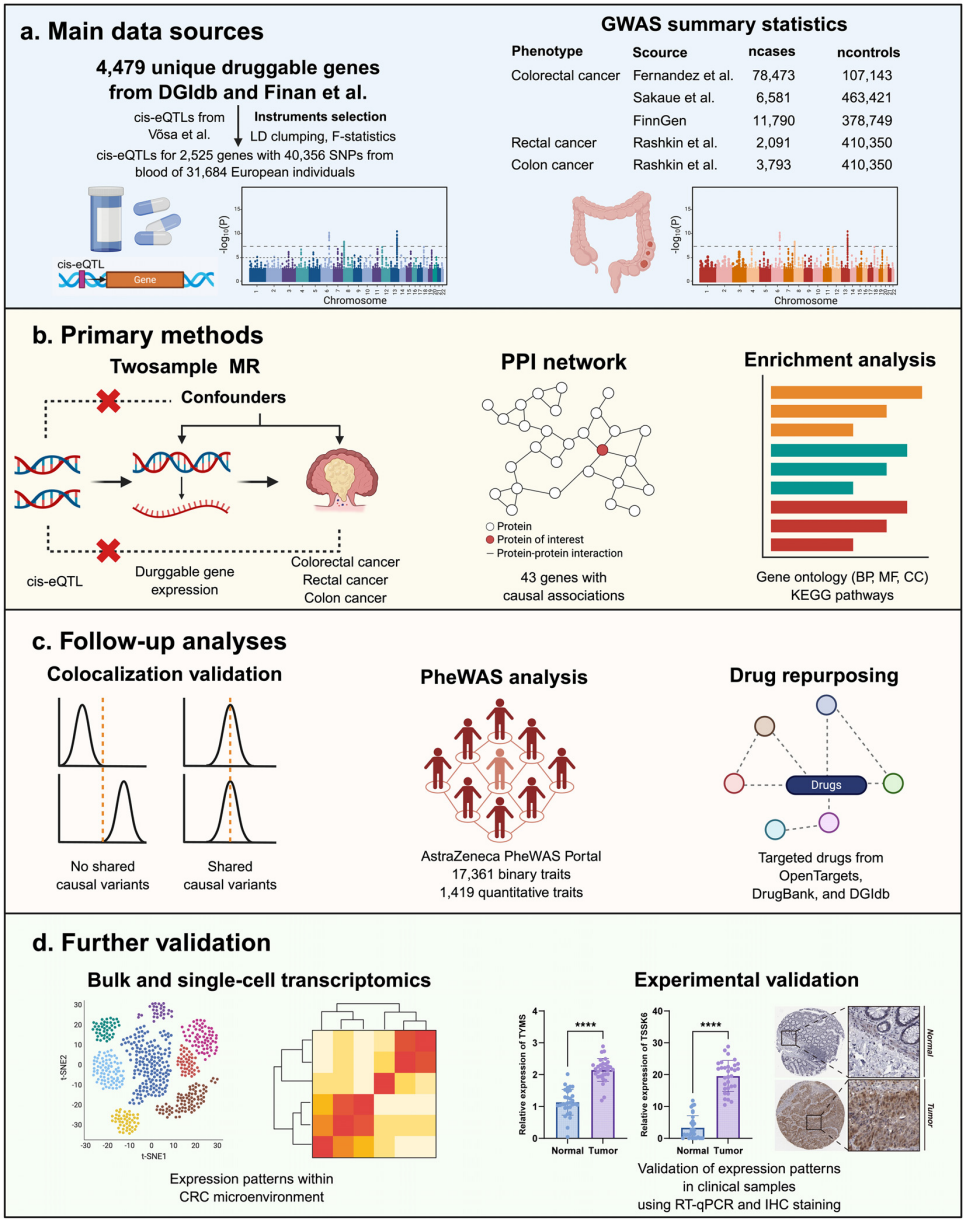
**(A–D)** Study design and workflow for identifying druggable gene targets associated with CRC.

Given these limitations, our study employs a Mendelian randomization (MR) approach to systematically identify potential drug targets for CRC. Unlike traditional observational studies, which are often confounded by environmental and lifestyle factors, MR leverages genetic variants as instrumental variables to infer causal relationships between gene expression and disease risk (18). This method reduces biases and enhances the robustness of target discovery, offering a powerful tool for drug repurposing and biomarker identification (19, 20). In our study, we integrate genome-wide association study (GWAS) data with expression quantitative trait loci (eQTL) analysis to prioritize druggable genes associated with CRC risk. By applying rigorous statistical criteria and sensitivity analyses, we aim to identify high-confidence targets that could be exploited for therapeutic intervention. Our findings provide a foundation for future preclinical and clinical studies, potentially paving the way for precision medicine strategies in CRC treatment (**Figure 1**).

## 2 Methods

### 2.1 Identification of druggable genes

To comprehensively identify potential therapeutic targets for CRC, we compiled a broad list of druggable genes, which encode proteins amenable to modulation by small molecules or biologics (21). Two primary resources were used, including the Drug–Gene Interaction Database (DGIdb v4.2.0) (22) and a comprehensive review of human druggable genes (21). These sources integrate data from drug-target databases, clinical studies, and chemical biology literature to define the druggable genome. From these databases, we curated a total of 4,479 unique druggable genes (**Supplementary Table S1**). This wide-scope inclusion strategy ensured that we captured both clinically validated targets and exploratory candidates relevant to complex diseases, including cancer. These genes were then used as input for subsequent eQTL and MR analyses to evaluate their causal relevance to CRC risk.

### 2.2 Acquisition of cis−eQTLs

Cis-eQTLs were defined as genetic variants located within 1 megabase (Mb) of the transcription start site of a gene and significantly associated with its expression (minor allele frequency > 0.01). We obtained cis-expression quantitative trait loci (cis-eQTLs) data from the eQTLGen Consortium (https://eqtlgen.org/), which provides large-scale eQTL information derived from blood samples of 31,684 individuals of European ancestry (23). This dataset includes expression data for 16,987 genes and over 30,000 significant cis-eQTLs. Among the 4,479 druggable genes initially identified, 2,525 had corresponding cis-eQTLs available in the eQTLGen dataset and were included in downstream MR analysis. This dataset served as the basis for selecting genetic instruments to evaluate causal relationships between gene expression and CRC risk.

### 2.3 Outcome data

Summary-level GWAS data for CRC were obtained from previously published meta-analyses and biobank studies, all based on individuals of European ancestry (24). The GWAS statistics could be further obtained from the GWAS catalog (https://www.ebi.ac.uk/gwas/home). The discovery analysis included 78,473 CRC cases and 107,143 controls (GCST90255675). Two replication datasets were used: one from Sakaue et al. (6,581 cases and 463,421 controls; GCST90018808) (25) and one from the FinnGen project (11,790 cases and 378,749 controls) (26). To explore site-specific effects, we also conducted stratified analyses using GWAS data for colon cancer (3,793 cases and 410,350 controls; GCST90011811) and rectal cancer (2,091 cases and 410,350 controls; GCST90011810) (27). All participants provided informed consent, and ethical approvals were obtained from the relevant institutional review boards. Detailed cohort characteristics are provided in **Table 1**.

**Table 1:** Information on the GWAS datasets analyzed in this study.

Trait	GWAS ID	Sample size	ncases	ncontrols	Ancestry
Colorectal cancer	GCST90255675	185,616	78,473	107,143	European
Colorectal cancer	GCST90018808	470,002	6,581	463,421	European
Colorectal cancer	C3_COLORECTAL_EXALLC	390,539	11,790	378,749	European
Rectal cancer	GCST90011810	412,441	2,091	410,350	European
Colon cancer	GCST90011811	414,143	3,793	410,350	European

### 2.4 Selection of instrumental variable

To ensure robust causal inference in MR, we applied strict criteria for selecting single nucleotide polymorphisms (SNPs) as instrumental variables (IVs) for each druggable gene. First, we extracted cis-eQTL SNPs with genome-wide significance (*P* < 5.0 × 10^-8^) (28) from the eQTLGen dataset, ensuring strong association with gene expression. To eliminate linkage disequilibrium (LD), SNPs were clumped using the 1000 Genomes Project (European population) reference panel, with an LD threshold of r^2^ < 0.1 and a clumping window of 10,000 kb (29). SNPs incompatible across exposure and outcome datasets were excluded. For palindromic SNPs, we inferred strand orientation based on allele frequencies or removed them if this information was unavailable. The F-statistic was calculated for each IV using the formula F = (N − k − 1)/k × [R^2^/(1 − R^2^)] to assess instrument strength (30). Only SNPs with F-statistics > 20 were retained to avoid weak instrument bias. After filtering, a total of 40,356 SNPs were selected as IVs corresponding to 2,525 druggable genes, forming the basis for downstream MR analysis (31). Details about the IVs are shown in **Supplementary Table S2**.

### 2.5 MR analysis

We conducted two sample MR analysis using the TwoSampleMR R package (version 0.6.6) to estimate the causal effects of gene expression on CRC, colon cancer, and rectal cancer risk. For genes with only a single valid instrumental SNP, we applied the Wald ratio method. When two or more independent SNPs were available, the inverse-variance weighted (IVW) method was used to derive overall causal estimates (32). To control for multiple hypothesis testing, we applied Bonferroni correction based on the total number of gene tested (33). Statistical significance thresholds were set at *P* < 2.02 × 10^-5^. This corrected threshold ensured robust identification of causal genes while minimizing the false discovery rate.

### 2.6 Sensitivity analysis

To assess the robustness of the MR results and detect potential violations of MR assumptions, we conducted a series of sensitivity analyses for genes with significant causal associations. For genes with multiple IVs, MR-Egger intercept tests were performed to evaluate the presence of horizontal pleiotropy, with *P* < 0.05 indicating potential bias (34). We also calculated Cochran’s Q statistic under both the IVW and MR-Egger models to assess heterogeneity among the SNP-specific estimates (35). Additionally, a leave-one-out analysis was carried out by iteratively excluding one SNP at a time to identify any disproportionately influential variants. Only genes that met all quality control criteria, including consistent effect directions across MR methods, non-significant MR-Egger intercepts, and stable leave-one-out results, were retained as high-confidence targets for further investigation.

### 2.7 Construction of protein-protein interaction network and enrichment analysis

To explore the functional interactions among genes identified by MR, we constructed a protein-protein interaction (PPI) network using the STRING database (https://string-db.org), limiting results to *“Homo sapiens”* and applying a minimum confidence score of 0.15. The network included only interactions supported by experimental or database evidence. To further investigate the biological relevance of these genes, we performed functional enrichment analysis using the clusterProfiler R package (36). Gene Ontology (GO) enrichment was used to categorize genes based on biological process (BP), cellular component (CC), and molecular function (MF) (37). In parallel, Kyoto Encyclopedia of Genes and Genomes (KEGG) pathway analysis was conducted to identify significantly enriched signaling pathways (38). Only terms and pathways with *P* < 0.05 were considered statistically significant. These analyses provided insights into the potential mechanisms through which the causal genes may influence CRC development and progression.

### 2.8 Colocalization analysis

Colocalization analysis was performed using the coloc R package (39) to determine whether gene expression and CRC risk share the same causal variant. SNPs were harmonized using the TwoSampleMR pipeline to ensure alignment between exposure and outcome datasets. Default prior probabilities were set to P1 = 1 × 10^-4^ (association with gene expression), P2 = 1 × 10^-4^ (association with CRC), and P12 = 1 × 10^-5^ (association with both). The posterior probabilities (PP) were used to evaluate five hypotheses, with strong colocalization defined as PP.H4/(PP.H3 + PP.H4) > 0.7 (40, 41), indicating a high likelihood that both traits are influenced by the same causal variant. Genes meeting this threshold were prioritized as high-confidence druggable targets.

### 2.9 Phenome−wide association analysis

To assess potential off-target effects and horizontal pleiotropy of the prioritized druggable genes, we conducted a phenome-wide association study (PheWAS) using the AstraZeneca PheWAS Portal (https://azphewas.com) (42). This platform includes genotype-phenotype associations derived from approximately 450,000 UK Biobank participants, covering ~15,500 binary and ~1,500 continuous phenotypes (43). For each candidate gene, we queried associated variants to identify significant relationships with traits unrelated to CRC. A significance threshold of *P* < 1 × 10–^6^ was applied, in accordance with the portal’s recommended cutoff for suggestive associations, to control for multiple testing and reduce false positives. This analysis provided insight into the broader phenotypic impact of each gene, helping to evaluate the safety and specificity of targeting these genes in CRC therapy.

### 2.10 Drug evaluation and repurposing

Given that most therapeutic agents target small-molecule proteins, we aimed to identify druggable targets among the 6 causal genes showing strong colocalization signals with CRC. To explore potential repurposing opportunities, we integrated our results with evidence from OpenTargets (44), DrugBank (45), and DGIdb (22). This integrative approach allowed us to prioritize candidate compounds, both approved and investigational, with favorable safety profiles that could be repositioned for CRC therapeutic applications.

### 2.11 Transcriptomic data analysis

The scRNA-seq data, comprising 33 samples from pre- and post-treatment tumor tissues and adjacent normal tissues of CRC patients, were retrieved from the GEO database (accession ID: GSE205506). We applied a strict quality control criteria to ensure data reliability. Cells were retained if they contained fewer than 20% mitochondrial gene content, expressed more than 200 genes, and had between 200 and 6000 detected genes, appearing in at least three cells. A total of 175,566 high-quality cells were selected for downstream analysis. To correct batch effects and improve clustering accuracy, we utilized the Harmony algorithm for data integration (46). The data was normalized using log-normalization, and the FindVariableFeatures function identified the top 2000 highly variable genes. Principal Component Analysis (PCA) was performed for dimensionality reduction, followed by soft k-means clustering using the Harmony package. Cells were then grouped into distinct clusters using the FindClusters function with a resolution of 0.6. Cell type annotation was conducted based on canonical marker genes, differential expression patterns, and known cellular profiles. Additionally, the expression patterns of potential targets were identified in bulk RNA-seq and scRNA-seq data. Bulk RNA-seq data, the TCGA-COAD cohort, was downloaded from UCSC Xena data portal (https://xena.ucsc.edu/). We first compared the expression levels of the targets between tumor and adjacent normal tissues. Subsequently, featureplots were utilized to visualize the expression patterns of target genes in CRC microenvironment. Additionally, we analyzed the association between the expression levels of target genes and key T cell exhaustion markers (PD-1, PD-L1, TIM-3) as well as the immune-suppressive cytokine IL-10 via GEPIA2 (http://gepia2.cancer-pku.cn/).

### 2.12 Real-time quantitative PCR

Paired tumor and adjacent normal tissues were collected from 31 patients with histologically confirmed CRC at Renji Hospital, Shanghai Jiao Tong University School of Medicine (Approval No.: KY2024-188-C). Tissue samples were immediately snap-frozen in liquid nitrogen and stored at -80°C until further processing. Total RNA was extracted using TRIzol reagent (Invitrogen) in accordance with the manufacturer’s instructions. Reverse transcription was performed using Superscript II reverse transcriptase (Invitrogen) and gene-specific primers. Real-time quantitative PCR (RT-qPCR) was conducted using the ABI Prism 7300 Sequence Detection System (Applied Biosystems) to assess the expression of LAMC1, TFRC, TNFSF14, PLK1, TYMS, TSSK6, and the reference gene GAPDH. Relative transcript levels were normalized to GAPDH expression using the relative standard curve method, following the manufacturer’s technical guidelines (Applied Biosystems). All data analysis adhered to the MIQE guidelines (47), ensuring experimental transparency and reproducibility. Detailed primer sequences were provided in **Supplementary Table S3**. The statistical differences were analyzed by one-way ANOVA and t-test using GraphPad Prism 10.1.2 (San Diego, CA, USA). *P* < 0.05 were considered significantly different.

### 2.13 Immunohistochemical staining

Immunohistochemical (IHC) images of both tumor and normal tissues were obtained from the Human Protein Atlas (HPA, https://www.proteinatlas.org/). However, IHC data of both normal and tumor tissues for TSSK6 were not available in the database and were therefore not included in the analysis.

## 3 Results

### 3.1 Identification of genes causally associated with CRC risk

**Figure 2:**
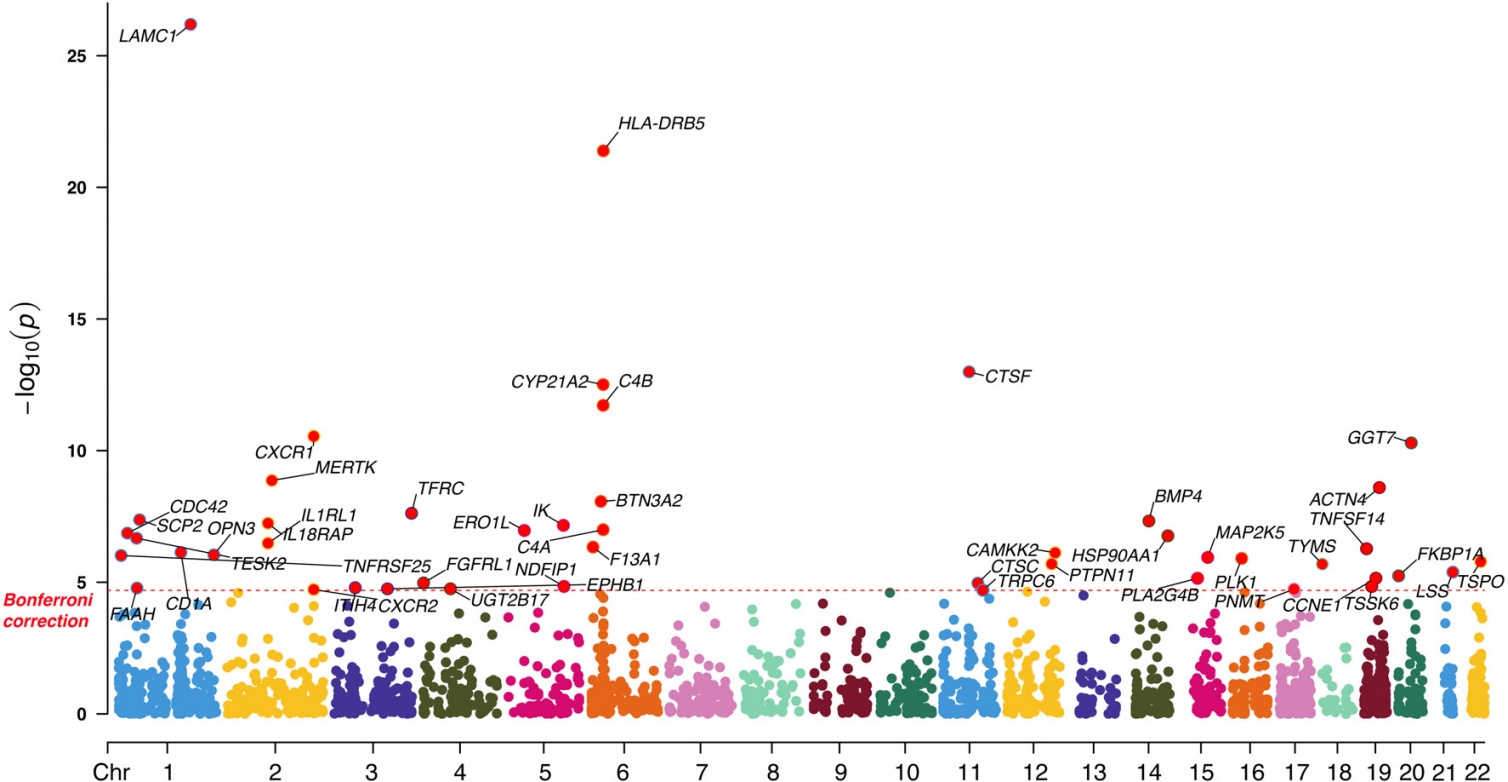
Manhattan plot illustrating the causal effect of significant druggable genes on CRC in the discovery cohort.

Using a two-sample MR framework, we systematically assessed the causal relationship between the expression of 2,525 druggable genes and CRC risk. After Bonferroni correction (*P* < 2.02 × 10^-5^), 47 genes demonstrated significant causal associations in the discovery cohort, including 45 via the IVW method and 2 via the Wald ratio method (**Figure 2**; **Supplementary Table S4**). To ensure robustness, we conducted sensitivity analyses, including MR-Egger intercept, Cochran’s Q test, and leave-one-out tests. Four genes (HLA-DRB5, CTSF, TSPO, and TRPC6) were excluded due to evidence of horizontal pleiotropy (*P* < 0.05). The remaining 43 genes passed all quality control criteria and were retained for further validation and downstream analyses. These results provide a high-confidence set of candidate genes potentially involved in CRC pathogenesis and highlight their potential as therapeutic targets.

### 3.2 Construction of PPI network and enrichment analysis

**Figure 3:**
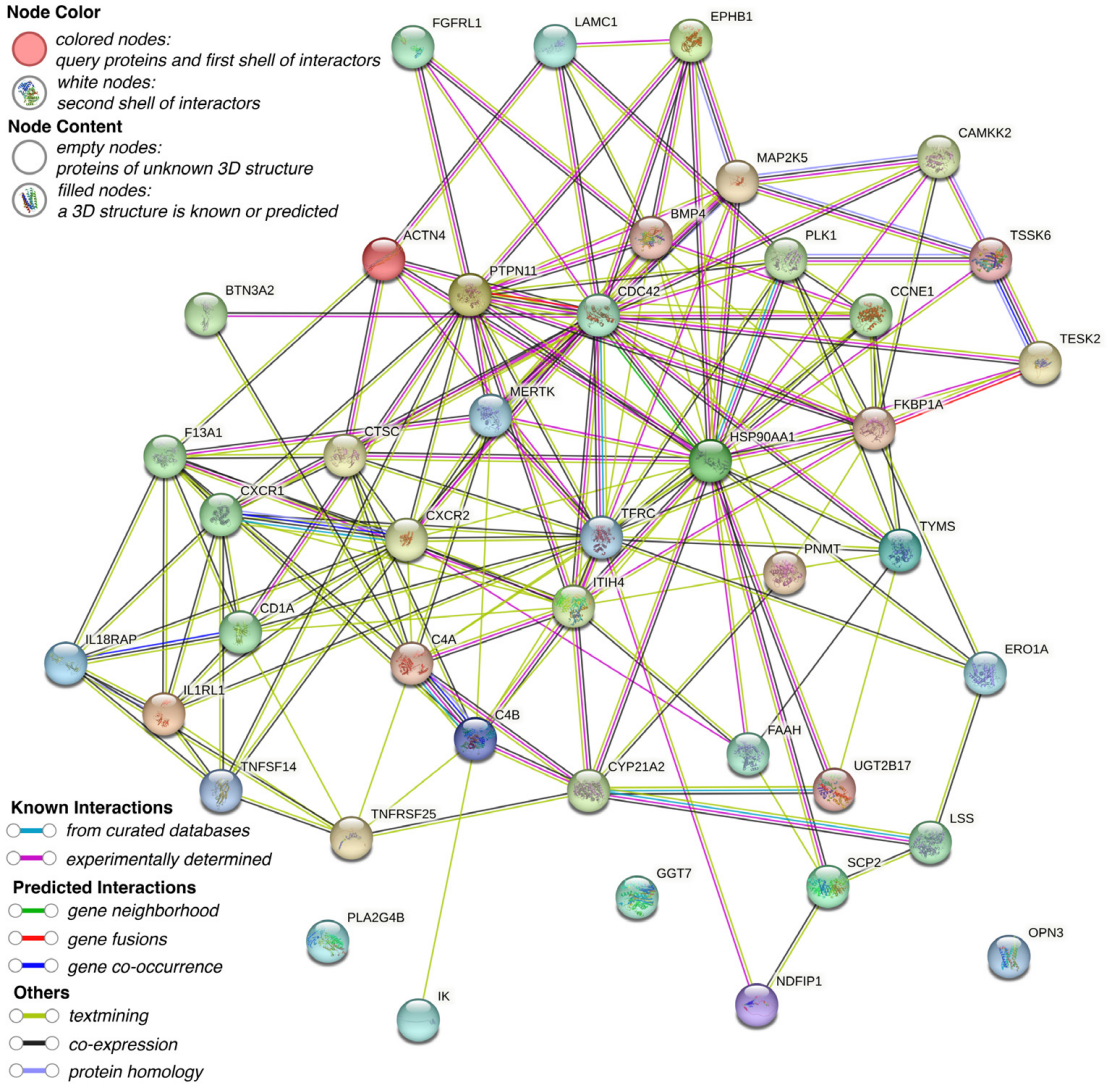
PPI network of CRC-associated genes constructed via STRING.

**Figure 4:**
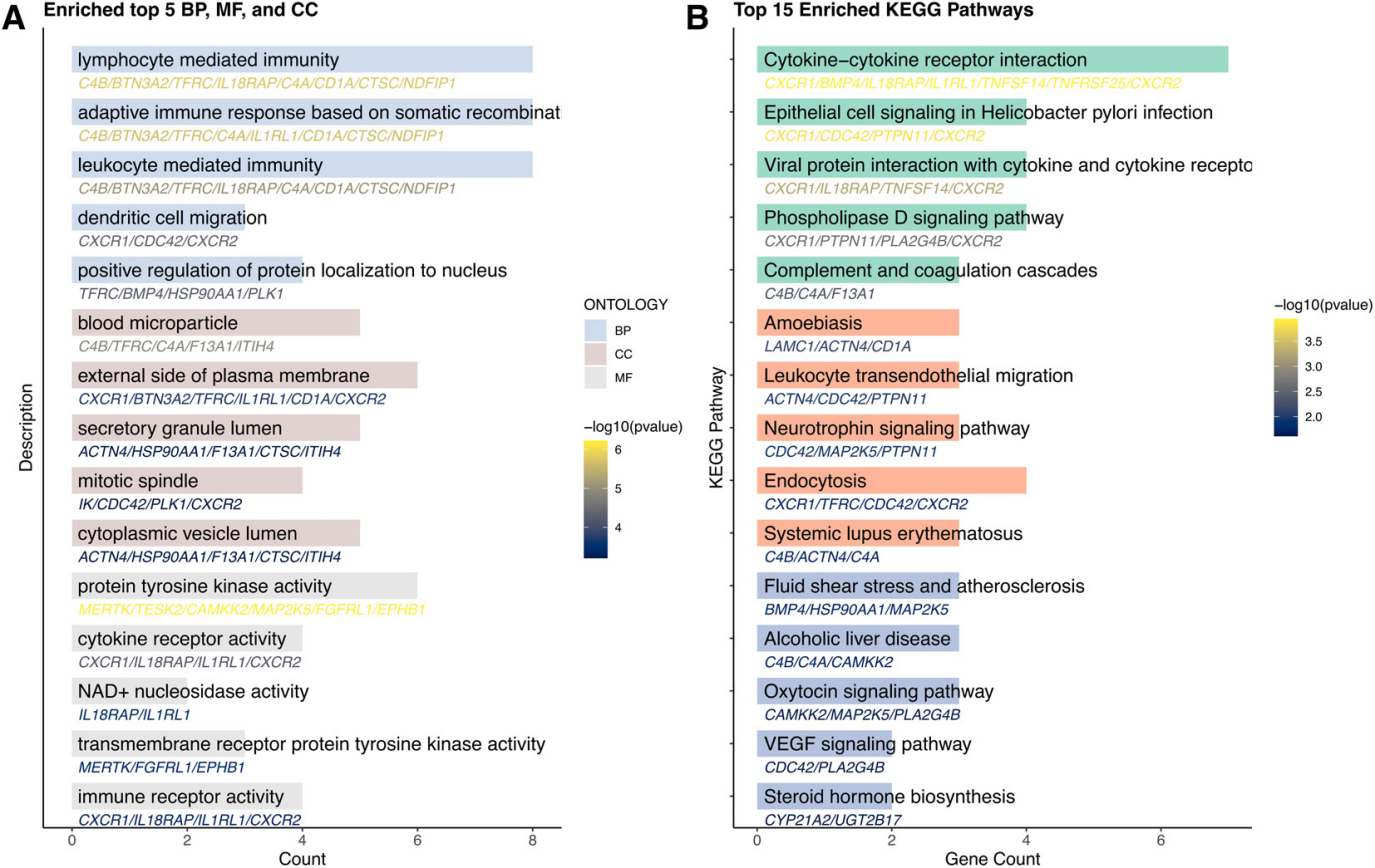
Results of enrichment analysis. GO **(A)** and KEGG **(B)** enrichment analyses of candidate genes.

To elucidate the functional relationships and interaction landscape among the genes causally linked to CRC, we constructed a PPI network using the STRING database. The network was restricted to interactions in *“Homo sapiens”* and filtered with a minimum interaction score of 0.15 to ensure confidence in the associations (**Figure 3**). The resulting network revealed a highly interconnected core, with genes such as PTPN11, CDC42, TFRC, HSP90AA1, and PLK1 serving as central hubs, suggesting key regulatory roles in CRC-related pathways. To further explore the biological relevance of these candidate genes, GO and KEGG pathway enrichment analyses were conducted using the R package clusterProfiler. GO analysis identified significant enrichment in terms associated with immune function and cell signaling, particularly lymphocyte-mediated immunity, dendritic cell migration, and protein localization to the nucleus (**Figure 4A**). Cellular component analysis highlighted localization to secretory granules, cytoplasmic vesicle lumens, and the external side of the plasma membrane, while molecular function analysis emphasized roles in protein tyrosine kinase activity and cytokine receptor activity. KEGG pathway enrichment revealed that the candidate genes were significantly associated with cancer-relevant signaling pathways (**Figure 4B**). The most enriched pathways included cytokine-cytokine receptor interaction, epithelial cell signaling in Helicobacter pylori infection, and leukocyte transendothelial migration. These findings underscore the potential involvement of the identified genes in inflammation, immune regulation, and tumor microenvironment remodeling-hallmarks of CRC development and progression.

### 3.3 Prioritizing 6 genes in colocalization analysis

To further refine the causal gene list and minimize potential false positives due to LD, we performed colocalization analysis on the 43 genes identified from MR. This approach assessed whether CRC-associated variants and gene expression signals shared the same causal variant. Six genes (TFRC, TNFSF14, LAMC1, PLK1, TYMS, and TSSK6) met this stringent criterion, indicating a high likelihood that gene expression and CRC risk are driven by the same genetic variants (**Table 2**). These genes were subsequently prioritized as high-confidence druggable targets. The remaining 37 genes, while suggestive, did not reach the colocalization threshold and were retained as secondary candidates for further evaluation. Sensitivity analyses supported the robustness of these six genes, showing no evidence of horizontal pleiotropy or significant heterogeneity (**Table 3**). Consistent effects across alternative MR models and leave-one-out analyses further confirmed the stability of these associations (**Supplementary Figures S1**, **S2**). These results highlight a subset of genes with both strong causal and colocalization evidence, warranting focused investigation in downstream validation and translational studies.

**Table 2:** Colocalization results of eQTLs for 6 genes with CRC-associated SNPs.

Gene	PP.H0	PP.H1	PP.H2	PP.H3	PP.H4	PP.H4/(PP.H3+PP.H4)
TFRC	0.00%	40.21%	0.00%	8.04%	51.75%	86.55%
TNFSF14	0.00%	52.22%	0.00%	4.75%	43.03%	90.05%
LAMC1	0.00%	0.01%	0.00%	9.14%	90.85%	90.86%
PLK1	0.00%	21.82%	0.00%	4.30%	73.88%	94.50%
TYMS	0.00%	59.42%	0.00%	11.42%	29.16%	71.85%
TSSK6	0.00%	30.87%	0.00%	15.54%	53.59%	77.52%

**Table 3:** Pleiotropy and heterogeneity test of the MR analysis.

Exposure	Outcome	Pleiotropy		Heterogeneity			
		Egger_intercept	*P*-value	IVW Q	*P*-value	MR Egger Q	*P*-value
TFRC	CRC	0.002	0.515	31.072	0.612	30.639	0.585
TNFSF14	CRC	-0.008	0.094	20.996	0.137	17.056	0.253
LAMC1	CRC	-0.006	0.267	97.985	0.001	95.875	0.001
PLK1	CRC	-0.001	0.929	7.382	0.287	7.369	0.195
TYMS	CRC	0.002	0.803	3.742	0.809	3.674	0.721
TSSK6	CRC	0.010	0.357	1.939	0.747	0.762	0.859

### 3.4 Replication and stratified analysis

**Figure 5:**
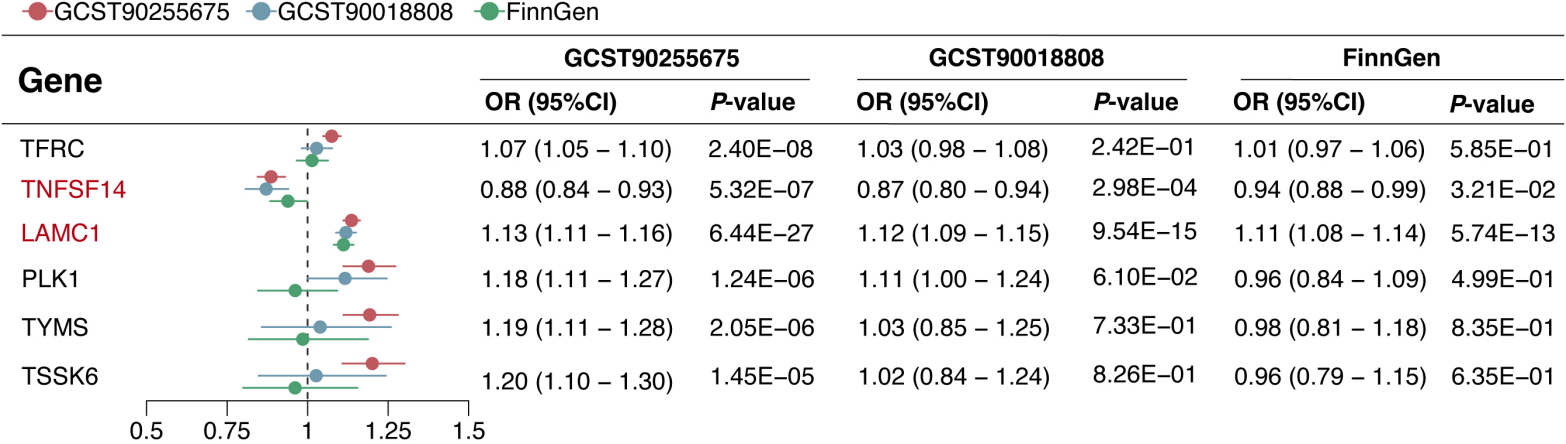
Causal effects of 6 candidate genes on CRC in the discovery and replication cohorts.

**Figure 6:**
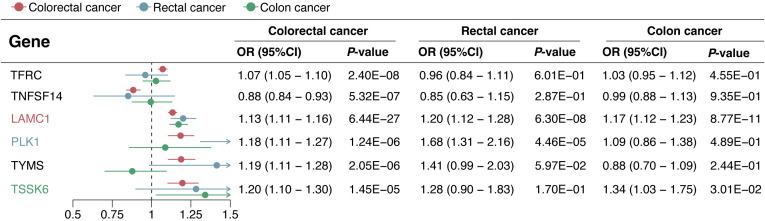
Causal effects of 6 candidate genes on CRC, rectal cancer, and colon cancer.

In the replication phase, among the 6 genes with significant colocalization evidence, 2 genes (LAMC1 and TNFSF14) were validated to be significant in all three replication cohorts (*P* < 0.05), and another 2 genes (TFRC and PLK1) were validated to be significant in at least one replication cohort (*P* < 0.05) (**Figure 5**). Additionally, the 6 genes with significant colocalization evidence were utilized for further stratified analysis in rectal cancer and colon cancer, respectively. In rectal cancer, two of the 6 genes (LAMC1 and PLK1) were identified with significant casual effect (*P* < 0.05) (**Figure 6**). In colon cancer, 2 of the 6 genes (LAMC1, and TSSK6) were identified with significant casual effect (*P* < 0.05) (**Figure 6**). Sensitivity analyses supported these findings, as no evidence of horizontal pleiotropy was detected for these genes (**Supplementary Tables S5**, **S6**). Consistent trends across four additional MR models and stability in the leave-one-out analysis confirmed the robustness of these findings (**Supplementary Figures S3**–**S6**).

### 3.5 PheWAS indicated no potential side effects of drugs targeting the 6 candidate genes

**Figure 7:**
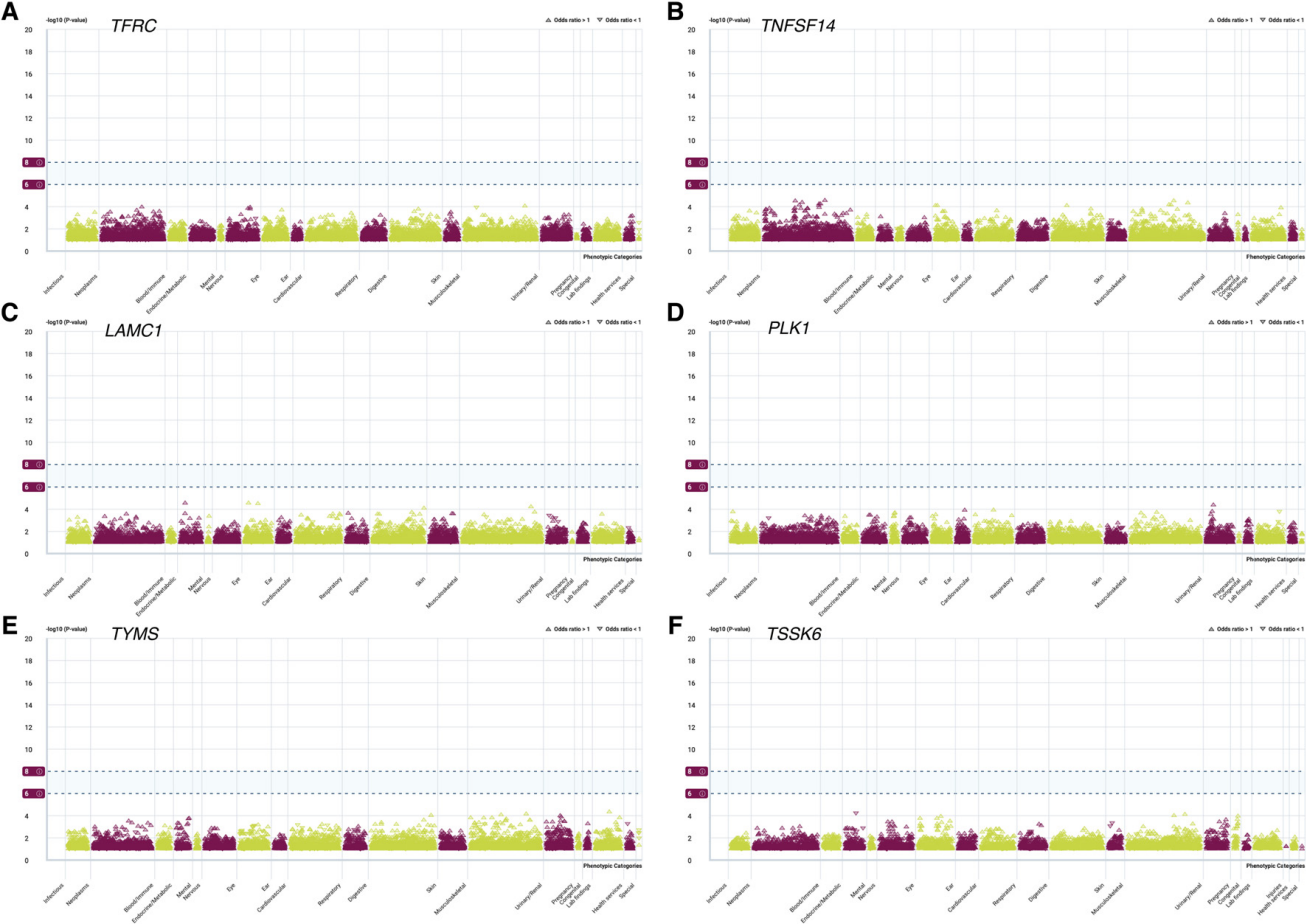
PheWAS analysis of associations between 6 candidate genes and various phenotypic categories. PheWAS analysis of TFRC **(A)**, TNFSF14 **(B)**, LAMC1 **(C)**, PLK1 **(D)**, TYMS **(E)**, and TSSK6 **(F)**.

The phenome-wide scan explored the relationships between potential drug targets and a variety of traits, providing valuable insights into possible side effects during drug development. This analysis revealed that none of the thirteen genes with strong colocalization evidence were linked to multiple phenotypes (*P* < 5 × 10^-8^) (**Figures 7A–F**).

### 3.6 Drug and compound prediction and existing drugs evaluation

To assess therapeutic potential, we investigated drugs targeting the six key genes (TFRC, TNFSF14, LAMC1, PLK1, TYMS, and TSSK6) using DGIdb, OpenTargets, and DrugBank. Several approved or investigational drugs were identified (**Table 4**). LAMC1 is targeted by agents like Ocriplasmin and Lanoteplase, mainly used for non-cancer conditions, but may be repurposed due to its role in the tumor microenvironment. TFRC, involved in iron metabolism, is already targeted by various approved iron supplements, and Pabinafusp alfa is under investigation for other diseases, showing potential for CRC applications. TNFSF14, a key immune regulator, is targeted by Baminercept, suggesting possible synergy with immunotherapies. PLK1 has multiple targeted drugs, including investigational anticancer agents like Volasertib and Onvansertib, with several approved drugs also showing potential off-target interactions. TYMS is already a standard chemotherapy target in CRC, with approved drugs such as Fluorouracil, Capecitabine, and Raltitrexed confirming its therapeutic relevance. These findings support drug repurposing strategies for CRC, potentially accelerating the development of targeted therapies.

**Table 4:** Available drugs targeting 6 genes retrieved from DGIdb, OpenTaregts, and DrugBank.

Gene	Drug	Indication	Trial status
LAMC1	Ocriplasmin	Symptomatic vitreomacular adhesion	Approved
	Lanoteplase	Myocardial infarction	Investigational
TFRC	Pabinafusp alfa	Mucopolysaccharidosis type 2	Investigational
	Ferric cation	Iron deficiency anemia	Approved
	Ferrous ascorbate	Iron deficiency anemia	Approved
	Ferrous fumarate, Ferrous gluconate, Ferrous glycine sulfate, Ferrous succinate, Iron	Iron deficiency; Iron deficiency anemia	Approved
	Ferric citrate	Control serum phosphorus levels; Iron supplement	Approved
TNFSF14	Baminercept	Rheumatoid arthritis	Investigational
PLK1	Lansoprazole	Acid reflux (heartburn); Stomach ulcers	Approved
	Simvastatin	Lower lipid levels; Reduce cardiovascular event risk	Approved
	Stavudine	HIV infection	Approved
	Disulfiram	Chronic alcoholism	Approved
	Succimer	Heavy metal poisoning	Approved
	Idarubicin	Acute myeloid leukemia (AML) in adults	Approved
	Topotecan	Ovarian cancer; small cell lung cancer; Cervical cancer	Approved
	Dipyridamole	Prevention of postoperative thromboembolic complications of cardiac valve replacement, and angina	Approved
	Acitretin	Severe psoriasis in adults	Approved
	Erythromycin	Treatment and prevention of a variety of bacterial infections	Approved
	Omeprazole	Improve the symptoms of heartburn; Treat related conditions such as ulcers, tissue damage and infection with H. pylori.	Approved
	Volasertib	Acute myeloid leukemia (AML); Myelodysplastic syndrome (MDS); Acute lymphoblastic leukemia (ALL); Various lymphomas (including T-cell and NK-cell types), leukemias (including erythroblastic and monocytic); Ovarian cancer; Non-small cell lung cancer (NSCLC); Other solid tumors	Investigational
	Onvansertib	Colorectal, prostate, pancreatic, and breast cancers; AML; Other neoplasms	Investigational
	TAK-960	Cancer	Terminated
	Cafusertib	AML; Neoplasm	Investigational
	BI-2536	AML	Investigational
	MK-1496	Neoplasm	Investigational
	BI 2536	Advanced or metastatic NSCLC	Investigational
	Fostamatinib	Chronic immune thrombocytopenia (ITP) after attempting one other treatment	Approved, Investigational
	Rigosertib	MDS, refractory anemia with excess of blasts (RAEB); Cancer; Hepatoma; Neoplasms.	Investigational
TYMS	ANX-510	Breast cancer; Colorectal cancer (CRC); Gall bladder cancer; Pancreatic cancer	Investigational
	Capecitabine	A variety of cancer types	Approved, Investigational
	Floxuridine	Liver metastases of gastrointestinal malignancy	Approved
	Fluorouracil	Skin cancer or other cancers	Approved
	Fosifloxuridine nafalbenamide	CRC	Investigational
	Gemcitabine	Ovarian cancer; NSCLC; Metastatic breast cancer; Pancreatic cancer	Approved
	Methotrexate	Ovarian cancer; NSCLC; Metastatic breast cancer; Pancreatic cancer.	Approved
	OSI-7904L	Gastric and/or gastroesophageal adenocarcinoma (A-G/GEJA)	Investigational
	Pemetrexed	Mesothelioma; NSCLC	Approved, Investigational
	Pralatrexate	Relapsed or refractory peripheral T-cell lymphoma	Approved, Investigational
	Raltitrexed	Malignant neoplasm of colon and rectum	Approved, Investigational
	Tegafur, Tegafur-uracil	Various cancers, such as stomach cancer and colon cancer.	Approved, Investigational
	Thymectacin	CRC	Investigational
	Trifluridine	Keratoconjunctivitis and epithelial keratitis caused by simplex virus; Certain types of metastatic gastrointestinal cancers	Approved, Investigational

### 3.7 Expression patterns of 6 targets within the CRC microenvironment

**Figure 8:**
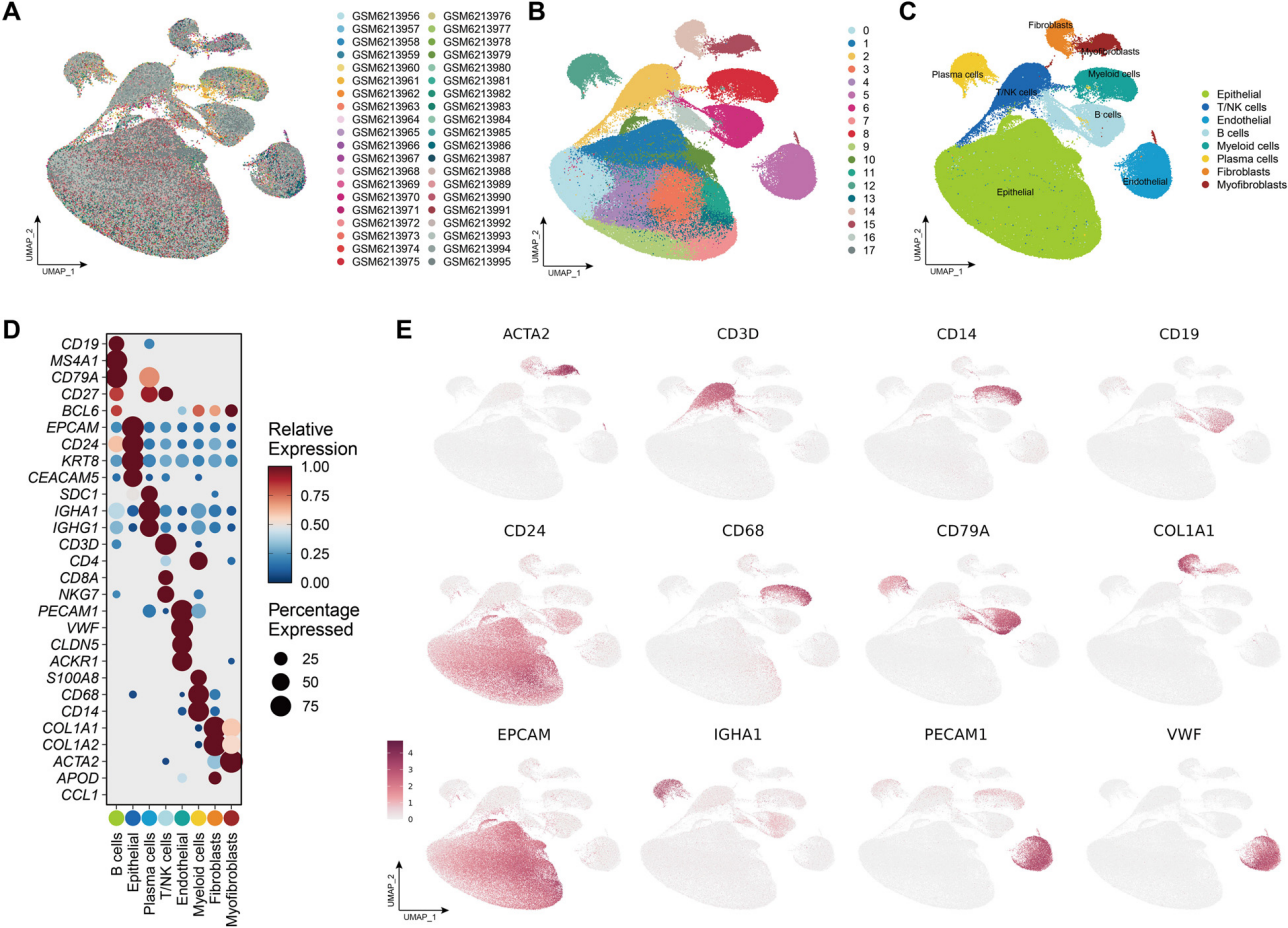
Single-cell landscape of the CRC tumor microenvironment. **(A)** UMAP plot showing the distribution of all samples included in the single-cell RNA-seq analysis. **(B)** Clustering of cells into distinct transcriptional states using unsupervised methods. **(C)** Annotation of major cell types, including Epithelial cells, T/NK cells (T cells and natural killer cells), Endothelial cells, B cells, Myeloid cells (monocytes, macrophages, dendritic cells), Plasma cells, Fibroblasts, and Myofibroblasts. **(D)** Dot plot showing the expression of canonical marker genes across annotated cell populations. Dot size represents the percentage of cells expressing each gene, while color reflects scaled average expression. **(E)** Feature plots visualizing the spatial expression patterns of selected marker genes across the UMAP space.

After applying the Harmony algorithm, the cellular distribution within each sample remained largely consistent, indicating the absence of significant batch effects among the samples, making them suitable for downstream analysis (**Figure 8A**; **Supplementary Figure S7**). A total of 18 distinct cell clusters were identified using a resolution parameter of 0.4 (**Figure 8B**). We reanalyzed 175,566 scRNA-seq cells spanning 26 samples, including primary tumor, adjacent normal tissues and PBMC samples. Based on classical marker genes, we successfully classified various cell populations, including epithelial cells, B cells, plasma cells, T/NK cells, myeloid cells, fibroblasts, myofibroblasts and endothelial cells (**Figure 8C**). The marker genes corresponding to each cell type displayed distinct expression patterns, reinforcing the accuracy of our cell annotation (**Figures 8D, E**).

**Figure 9:**
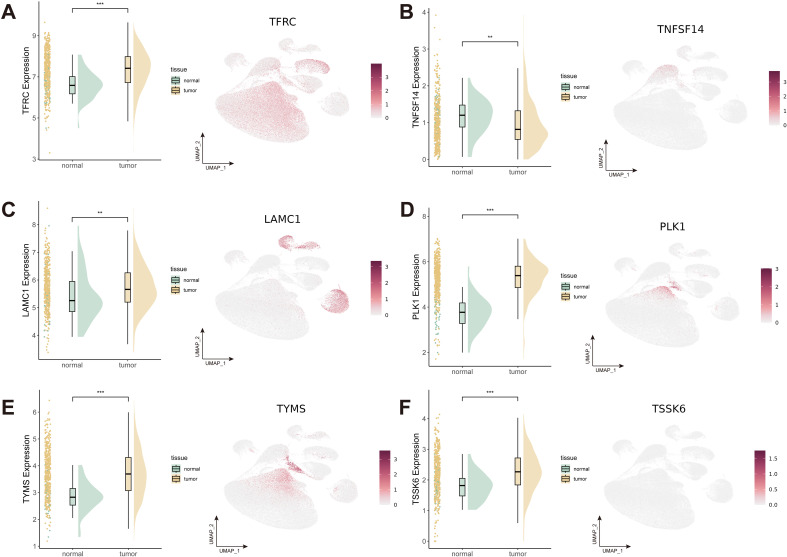
Expression patterns of 6 candidate genes with strong colocalization evidence. **(A-F)** Expression patterns of TFRC **(A)**, TNFSF14 **(B)**, LAMC1 **(C)**, PLK1 **(D)**, TYMS **(E)**, and TSSK6 **(F)**. **P < 0.01; ***P < 0.001.

Detailed information on the expression levels of the 6 candidate genes is presented, along with information regarding the different expression patterns within tumor and normal samples (**Figures 9A–F**). The expression levels of TFRC, LAMC1, PLK1, TYMS, and TSSK6 were significantly higher in tumor tissues while TNFSF14 exhibited higher expression levels in normal tissues. Notably, LAMC1 showed high expression levels in endothelial cells, fibroblasts and myofibroblasts, and TFRC in epithelial cells. The correlation analysis of the expression levels of target genes with key T cell exhaustion markers (PD-1, PD-L1, TIM-3) and the immunosuppressive cytokine IL-10 demonstrated that TNFSF14, LAMC1, and TYMS were significantly and positively associated with these immune markers (**Supplementary Figure S8**).

### 3.8 Validation of expression patterns of 6 candidate genes

**Figure 10:**
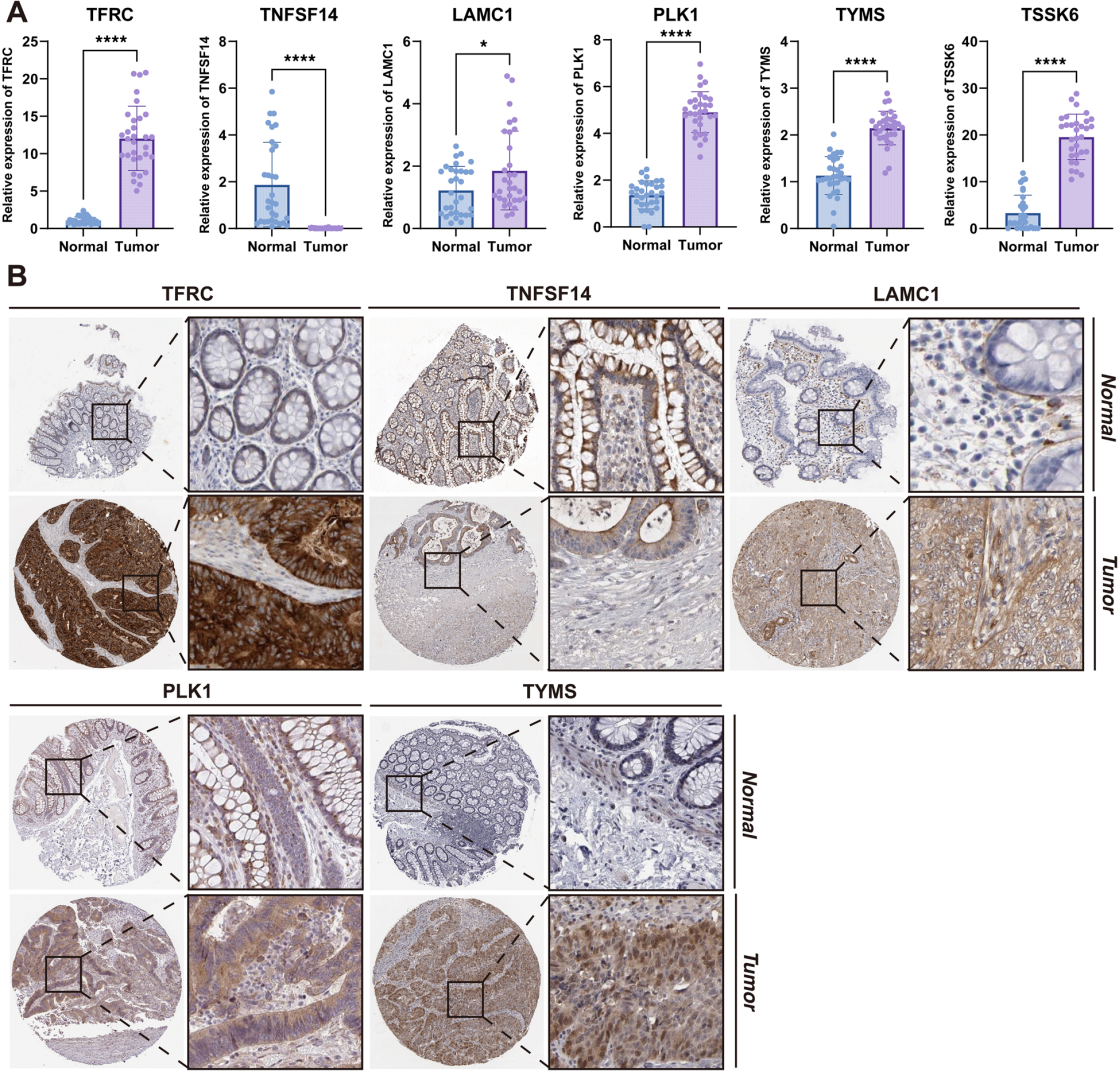
Experimental validation of gene expression by RT-qPCR and IHC. **(A)** RT-qPCR quantification of gene expression (TNFSF14, LAMC1, PLK1, TYMS, and TSSK6) in paired tumor and adjacent normal tissues from 31 CRC patients. Data presented as mean ± SD; significance determined by paired t-test (*P* < 0.05). **(B)** IHC images showing protein expression patterns. *P < 0.05; ****P < 0.0001.

We performed RT-qPCR to validate the results of our previous analyses, where consistent expression trends were observed (**Figure 10A**). Specifically, TFRC, PLK1, TYMS, and TSSK6 were remarkably upregulated in tumor tissues compared to normal controls (*P* < 0.0001). Similarly, LAMC1 showed a significant increase (*P* < 0.05), while TNFSF14 was significantly downregulated in tumor tissues (*P* < 0.0001). Consistent with these findings, the IHC staining results of the five targets demonstrated similar trends at the protein level (**Figure 10B**). Strong immunoreactivity of TFRC, LAMC1, PLK1, and TYMS was observed in tumor tissues, whereas weak or no staining was observed in normal tissues. TNFSF14 showed similar staining intensities in tumor and normal tissues.

## 4 Discussion

CRC remains a major global health challenge, with high morbidity and mortality despite advancements in screening and treatment strategies (1). Current treatment modalities, including chemotherapy, targeted therapy, and immunotherapy, have improved patient outcomes but are often limited by drug resistance and adverse effects (7). As precision oncology continues to evolve, the identification of novel drug targets is crucial for the development of more effective therapies (48, 49). Our study utilized a comprehensive approach combining MR, colocalization analysis, bulk and single-cell transcriptomics to systematically identify and validate potential druggable genes in CRC. We identified 6 candidate genes (TFRC, TNFSF14, LAMC1, PLK1, TYMS, and TSSK6) with strong evidence supporting their role in CRC pathogenesis and druggability. Additionally, through drug-gene interaction analysis using OpenTargets and DrugBank, we identified several existing drugs that may be repurposed for CRC treatment. These findings provide valuable insights into the genetic basis of CRC and highlight promising therapeutic opportunities.

LAMC1 (Laminin Subunit Gamma 1) is a key component of the extracellular matrix (ECM) and plays a crucial role in tumor progression, invasion, and metastasis by modulating cell adhesion, migration, and epithelial-to-mesenchymal transition (EMT) (50). Studies suggest that LAMC1 overexpression in CRC enhances tumor cell survival and chemoresistance by activating integrin/FAK signaling pathways. Given its role in ECM remodeling, targeting LAMC1 has the potential to disrupt malignancy-stromal interactions and prevent CRC metastasis.

TFRC (Transferrin Receptor) is essential for iron uptake and is highly expressed in rapidly proliferating tumor cells. Its overexpression in CRC promotes tumor growth by increasing intracellular iron levels, leading to enhanced DNA synthesis and oxidative metabolism (51–53). Iron metabolism dysregulation is a hallmark of cancer, and TFRC-targeting therapies, such as TfR1 antibodies or ferroptosis-inducing agents, have been explored in preclinical models.

TNFSF14, also known as LIGHT, is a member of the tumor necrosis factor (TNF) superfamily that plays a pivotal role in modulating anti-tumor immune responses. LIGHT engages two key receptors, HVEM (Herpesvirus Entry Mediator) and LTβR (Lymphotoxin Beta Receptor), to stimulate T cell proliferation, activation, and recruitment within the tumor microenvironment (TME) (54). Mechanistically, LIGHT–HVEM signaling has been shown to enhance T cell receptor–mediated activation, increase IFN-γ secretion, and support the effector function and persistence of cytotoxic CD8^+^ T cells (55). LIGHT–LTβR engagement promotes the formation of tertiary lymphoid structures (TLSs), improves vascular normalization, and enhances the infiltration of immune cells into the tumor core, thereby fostering a more permissive and immunologically active TME (56). In CRC, elevated LIGHT expression has been associated with enhanced infiltration of cytotoxic CD8^+^ T cells and a more favorable immune phenotype (57–59). Notably, TNFSF14 expression was significantly downregulated in tumor tissues compared to adjacent normal tissues, suggesting that CRC may suppress this pathway to evade immune surveillance. Given its role in activating lymphocytes, this pattern supports the immunostimulatory function of LIGHT and underscores its therapeutic potential in restoring anti-tumor immunity. However, the function of LIGHT can be context-dependent and subject to modulation by immunosuppressive signals within the TME. Specifically, TGF-β, PD-1/PD-L1, and CTLA-4 pathways may attenuate LIGHT-mediated immune activation by suppressing effector T cell responses or promoting regulatory T cell (Treg) expansion. Emerging evidence suggests that co-targeting LIGHT and immune checkpoints could synergistically overcome tumor-induced immune evasion (60). For instance, combination therapies using LIGHT agonists with anti-PD-1 or anti-CTLA-4 antibodies have shown enhanced tumor clearance in preclinical models by reinvigorating exhausted T cells and promoting durable anti-tumor immunity (61, 62). Moreover, mRNA-based approaches delivering TNFSF14 directly into dendritic cells or effector T cells may further amplify its immunostimulatory effect and help reshape an immune “cold” tumor into a “hot” one, rendering CRC more responsive to immune checkpoint blockade (62). These findings suggest that LIGHT may act as a key immunologic amplifier, whose effects are shaped by the broader signaling landscape of the TME, and that it holds promise as a combinatorial target for precision immunotherapy in CRC.

PLK1 is a serine/threonine kinase that plays a pivotal role in regulating mitotic progression (63). Dysregulation of PLK1 is highly frequent in various malignancies and is correlated with poor prognosis in many cancers (64). Previous studies have shown that PLK1 is overexpressed in CRC tumors compared with normal tissue and which is correlated with disease progression, including adverse invasion, metastasis, and prognosis (65–67). Notably, PLK1 contributes to the DNA damage response (DDR) and mitotic checkpoint activation, allowing CRC cells to survival despite of genotoxic stress. Therapeutically, PLK1 inhibitors such as Volasertib (BI 6727) and Onvansertib (NMS-P937) have demonstrated potential in preclinical and clinical studies (68–70). Onvansertib, in particular, is being investigated for its efficacy in KRAS-mutant CRC, where it enhances the response to chemotherapy (70). Additionally, CRISPR/Cas9-mediated PLK1 knockout or RNA interference (RNAi) approaches could further suppress tumor growth and increase sensitivity to DNA-damaging agents, providing a potential avenue for combination therapies.

TYMS encodes thymidylate synthase, a crucial enzyme involved in DNA synthesis and repair. It catalyzes the conversion of deoxyuridine monophosphate (dUMP) to deoxythymidine monophosphate (dTMP), which is essential for nucleotide biosynthesis. Overexpression of TYMS has been identified as a major mechanism of resistance to 5-fluorouracil (5-FU)-based chemotherapy in CRC, reducing treatment efficacy (71, 72). Additionally, high TYMS levels can activate the mTOR signaling pathway, further promoting tumor proliferation. Further, Martinez-Balibrea et al. found TYMS polymorphisms influence on tumor response and toxicities derived from irinotecan plus 5-fluorouracil treatment in CRC patients, and proposed a genetic-based algorithm to optimize treatment individualization (73). To overcome TYMS-related resistance, targeted TYMS inhibitors such as Raltitrexed have been developed as alternatives to 5-FU (74). Moreover, CRISPR/Cas9-mediated TYMS knockout has been proposed as a strategy to increase tumor susceptibility to fluoropyrimidine-based treatments (75). Another promising approach is the use of microRNA (miRNA) therapeutics, such as miR-192 and miR-215, which have been shown to downregulate TYMS expression and restore chemosensitivity in CRC cells (76, 77).

Our findings also uncovered TSSK as a novel and promising therapeutic target in CRC, which appear to contribute to chemoresistance. TSSK6 (Testis-Specific Serine/Threonine Kinase 6) is primarily expressed in the reproductive system but has been detected in various cancers, including CRC. Although its role in CRC remains poorly understood, emerging evidence suggests that TSSK6 may contribute to tumor progression through cell cycle regulation and chemoresistance (78). Inhibiting TSSK6 could provide a novel approach to sensitizing CRC cells to chemotherapy. Future studies should explore CRISPR-mediated TSSK6 deletion to evaluate its impact on tumor growth and drug resistance.

In comparison with previous CRC biomarker discovery studies, our integrative approach offers several distinct advantages. Traditional omics-based screens have often focused on differential gene expression or somatic mutation profiling alone, which, while informative, may not establish causal relationships between gene function and CRC pathogenesis. For example, large-scale transcriptomic analyses such as The Cancer Genome Atlas (TCGA) have identified numerous dysregulated genes in CRC, but many lack functional validation or clinical translatability due to confounding factors and tumor heterogeneity. Similarly, proteomic and epigenetic studies have proposed novel targets, yet few have progressed to actionable therapies due to challenges in target prioritization. In contrast, our study leverages a MR framework combined with colocalization analysis to infer causal gene-disease relationships, reducing confounding and increasing biological plausibility. Furthermore, by integrating bulk and single-cell RNA-seq data, we not only identify gene targets but also precisely map their expression across distinct cellular compartments in the tumor microenvironment. This spatial resolution is often absent in previous screens and enables more accurate prediction of therapeutic relevance, target accessibility, and potential resistance mechanisms. Additionally, our use of PheWAS to evaluate off-target effects provides a safety-centric perspective rarely addressed in omics studies. Together, these strengths highlight the novelty and translational relevance of our prioritized targets, which exhibit strong genetic associations, therapeutic tractability, and distinct expression patterns. These features not only distinguish them from previously reported candidates but also suggest their potential utility as biomarkers for patient stratification in future clinical trials, enabling more precise selection of individuals likely to benefit from targeted or immunotherapeutic interventions.

Despite the strengths of our integrative framework, several important limitations must be acknowledged. Specifically, both the eQTL and GWAS datasets employed in this study were derived entirely from individuals of European ancestry. This lack of population diversity may restrict the transferability of our findings to non-European populations, as allele frequencies, LD structures, and gene-environment interactions can differ substantially across ethnic groups. Therefore, future studies incorporating multi-ethnic cohorts, including Asian, African, and admixed populations, are essential to validate the identified targets and ensure their generalizability and clinical relevance in a globally diverse setting. Second, we focused on cis-eQTLs, which, while minimizing pleiotropy, restricted the detection of trans-regulatory effects and precluded bidirectional MR analyses, such as those assessing CRC-related gene expression changes. Third, transcriptomic data do not fully capture protein-level dynamics, as post-transcriptional regulation and protein degradation can affect gene function (79). Further validation using proteomics and CRISPR-based functional studies is essential to confirm the biological relevance of our targets. Fourth, while we identified several promising druggable genes and matched them to known compounds, preclinical validation, such as *in vivo* efficacy, toxicity assessment, and combination therapy testing, is necessary to advance these findings toward clinical application. Additionally, while we observed the expression of candidate genes in broader immune and stromal compartments, a more detailed investigation into their enrichment within specific immunosuppressive populations was beyond the scope of this work. Future studies incorporating higher-resolution cell-type classification and functional validation will be important to further elucidate the roles of these genes in immune evasion. Finally, while our analysis identified druggable targets in CRC, we were unable to stratify samples by key clinical or molecular subtypes such as MSI status or consensus molecular subtypes (CMS), due to the lack of subtype annotation in the available dataset. Given the well-established molecular and immunological heterogeneity of CRC, such stratification may significantly influence the expression and therapeutic relevance of candidate genes. Future studies incorporating subtype-specific validation will be essential to refine the clinical utility of our findings and to enable more precise immunotherapeutic strategies tailored to distinct CRC subpopulations.

## 5 Conclusion

This integrative genomic and single-cell framework identified six high-confidence druggable genes (LAMC1, TFRC, TNFSF14, PLK1, TYMS, and TSSK6) with potential therapeutic relevance in CRC. By combining MR, colocalization, transcriptomics, and drug-gene interaction analysis, we systematically prioritized targets with strong genetic evidence, cell-specific expression, and minimal predicted off-target effects. Several of these genes are already linked to approved or investigational compounds, offering opportunities for drug repurposing and precision treatment strategies. While further functional and preclinical validation is needed, our findings provide a robust foundation for advancing targeted therapies in CRC and highlight the utility of integrative omics approaches in oncology drug discovery.

## Data Availability

The original contributions presented in the study are included in the article/**Supplementary Material**. Further inquiries can be directed to the corresponding authors.
