# Supplementary material for: Integrative genomic and single-cell framework identifies druggable targets for colorectal cancer precision therapy
Source: Front Immunol. 2025 May 27;16:1604154. doi: 10.3389/fimmu.2025.1604154 (PMC12149120; doi:10.3389/fimmu.2025.1604154)
Supplement: Supplementary file 1 [file DataSheet1.docx]

***Supplementary Materials***

**Fig. S1** Scatter plots of the causal relationship between 6 genes with significantly strong colocalization and CRC.

**Fig. S2** Leave-one-out sensitivity analysis for the causal relationship between 6 genes with significantly strong colocalization and CRC.

**Fig. S3** Scatter plots of the causal relationship between 2 significant genes and rectal cancer.

**Fig. S4** Leave-one-out sensitivity analysis for the causal relationship between 2 significant genes and rectal cancer.

**Fig. S5** Scatter plots of the causal relationship between 2 significant genes and colon cancer.

**Fig. S6** Leave-one-out sensitivity analysis for the causal relationship between 2 significant genes and colon cancer.

**Fig. S7** UMAP plots comparing the cell clustering before and after batch correction. (A) Cell clustering before batch correction. (B) Cell clustering after batch correction.

**Fig. S8** Correlation analysis regarding the expression levels of target genes and key T cell exhaustion markers (PD-1, PD-L1, TIM-3) as well as the immune-suppressive cytokine IL-10. (A) TFRC vs. CD274. (B) TFRC vs. CD279. (C) TFRC vs. HAVCR2. (D) TFRC vs. IL-10. (E) TNSF14 vs. CD274. (F) TNFSF14 vs. CD279. (G) TNFSF14 vs. HAVCR2. (H) TNFSF14 vs. IL-10. (I) LAMC1 vs. CD274. (J) LAMC1 vs. CD279. (K) LAMC1 vs. HAVCR2. (L) LAMC1 vs. IL-10. (M) PLK1 vs. CD274. (N) PLK1 vs. CD279. (O) PLK1 vs. HAVCR2. (P) PLK1 vs. IL-10. (Q) TYMS vs. CD274. (R) TYMS vs. CD279. (S) TYMS vs. HAVCR2. (T) TYMS vs. IL-10. (U) TSSK6 vs. CD274. (V) TSSK6 vs. CD279. (W) TSSK6 vs. HAVCR2. (X) TSSK6 vs. IL-10.


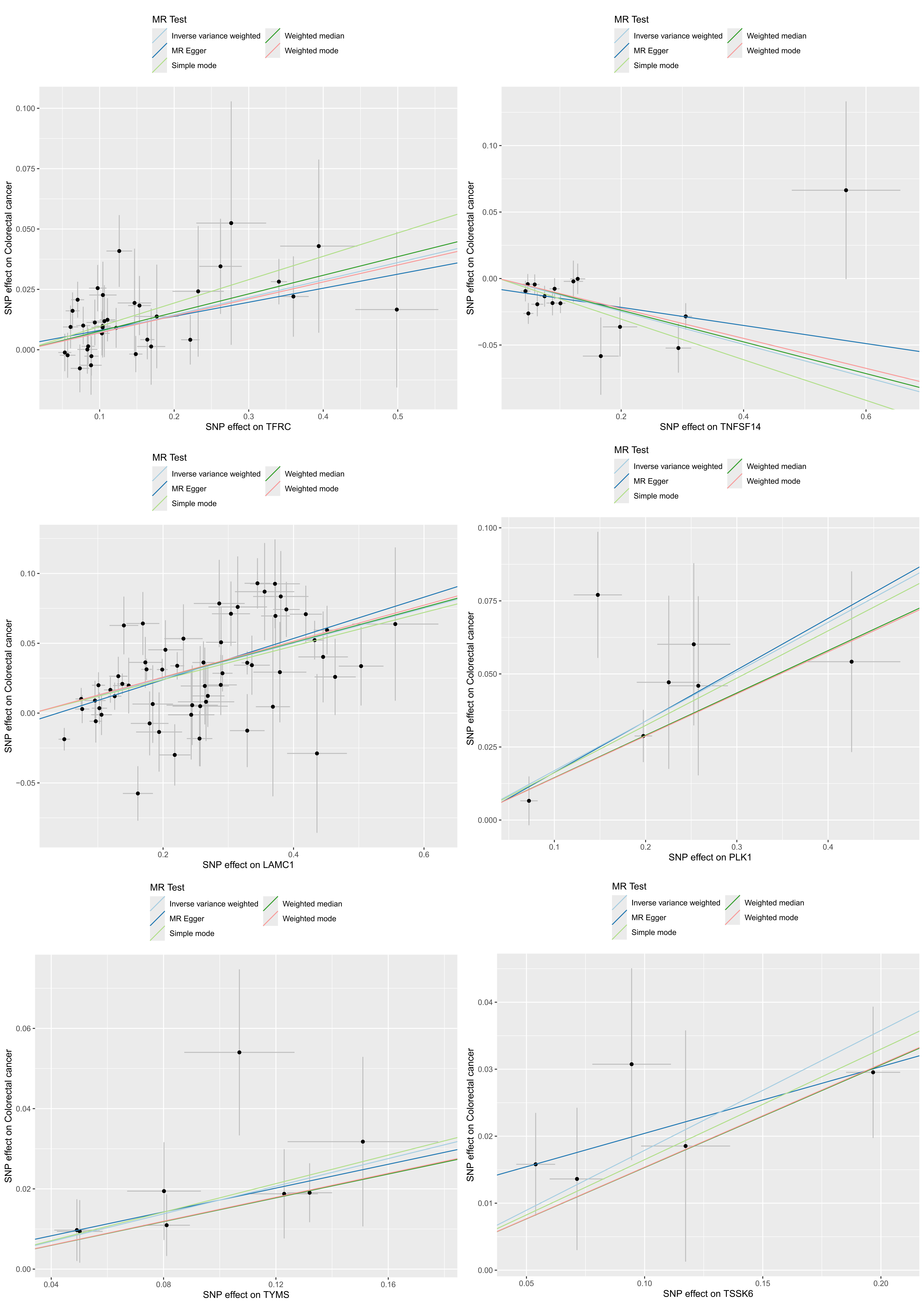


**Fig. S1** Scatter plots of the causal relationship between 6 genes with significantly strong colocalization and CRC.


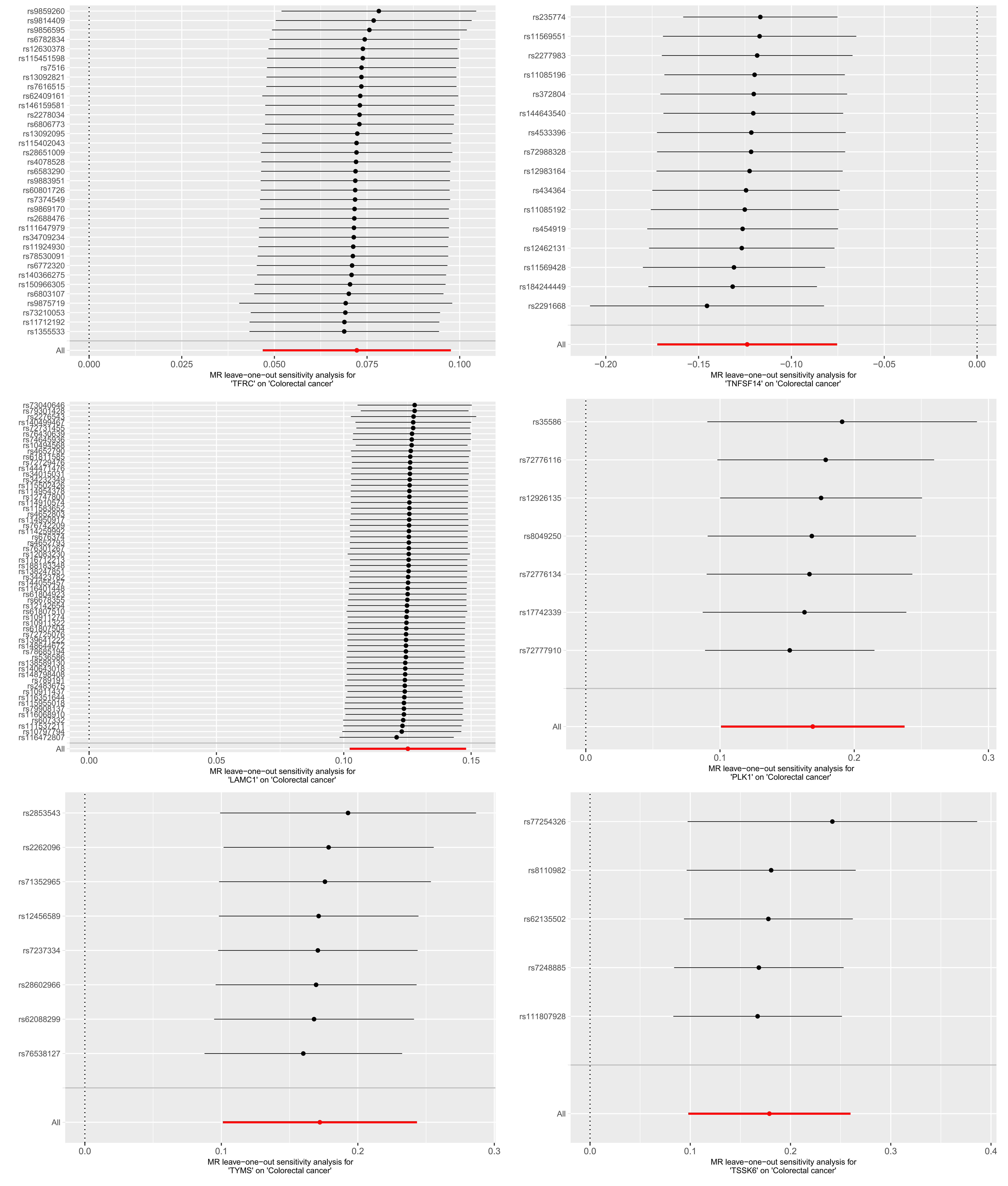


**Fig. S2** Leave-one-out sensitivity analysis for the causal relationship between 6 genes with significantly strong colocalization and CRC.


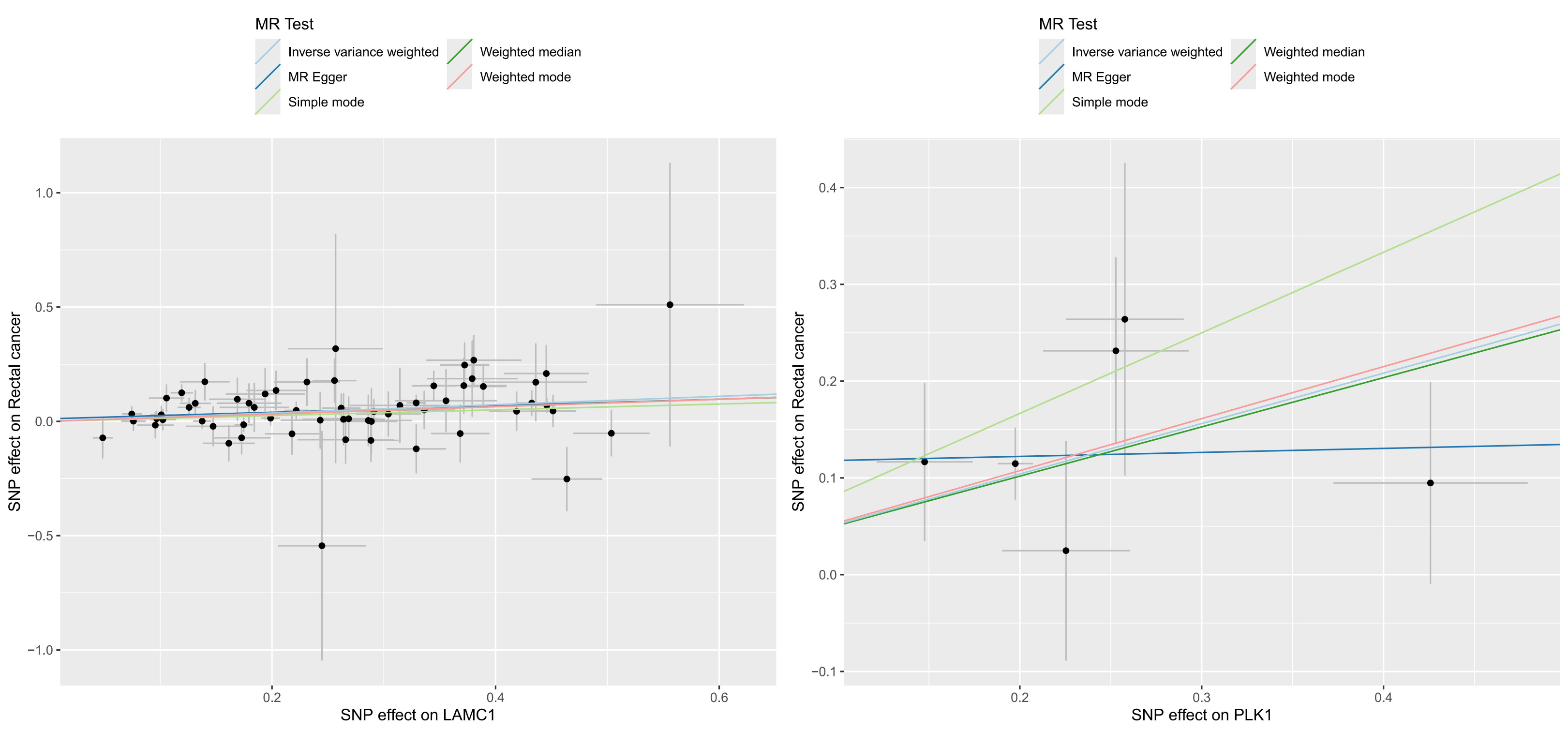


**Fig. S3** Scatter plots of the causal relationship between 2 significant genes and rectal cancer.


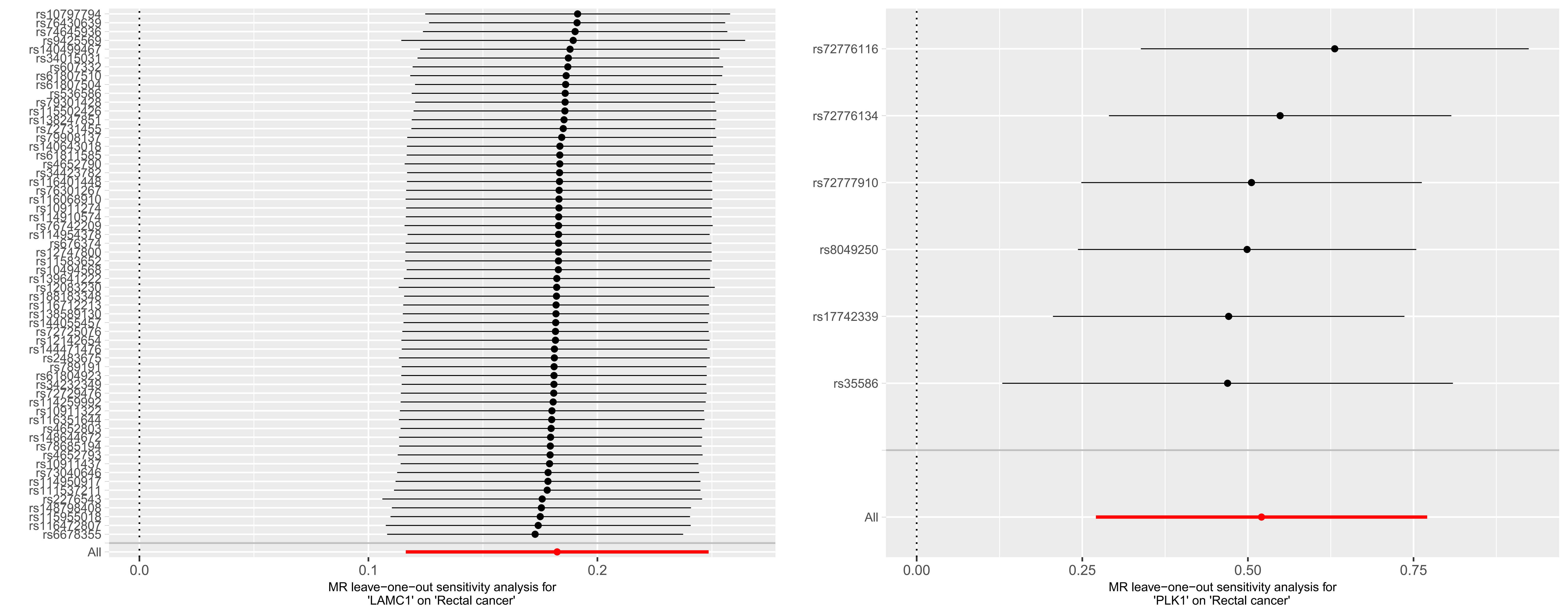


**Fig. S4** Leave-one-out sensitivity analysis for the causal relationship between 2 significant genes and rectal cancer.


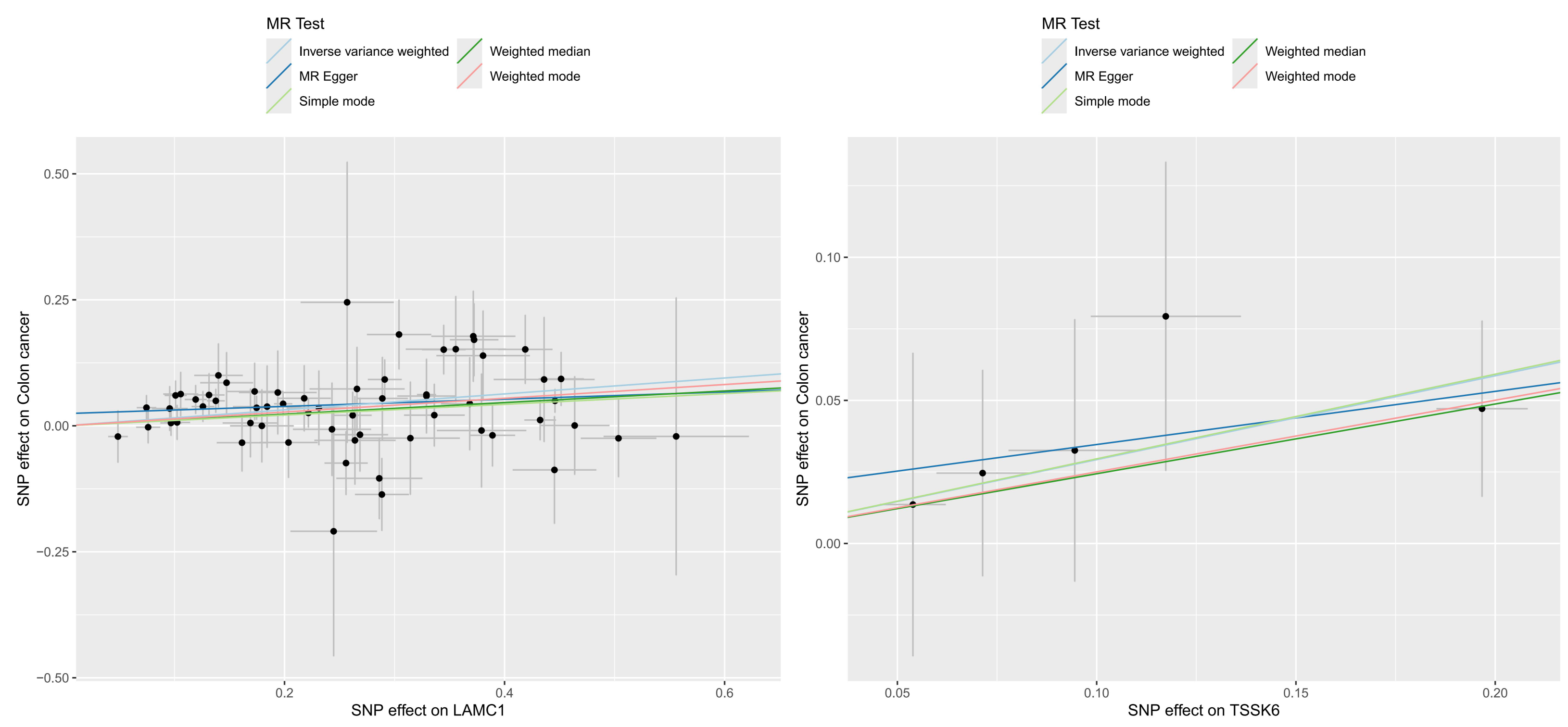


**Fig. S5** Scatter plots of the causal relationship between 2 significant genes and colon cancer.


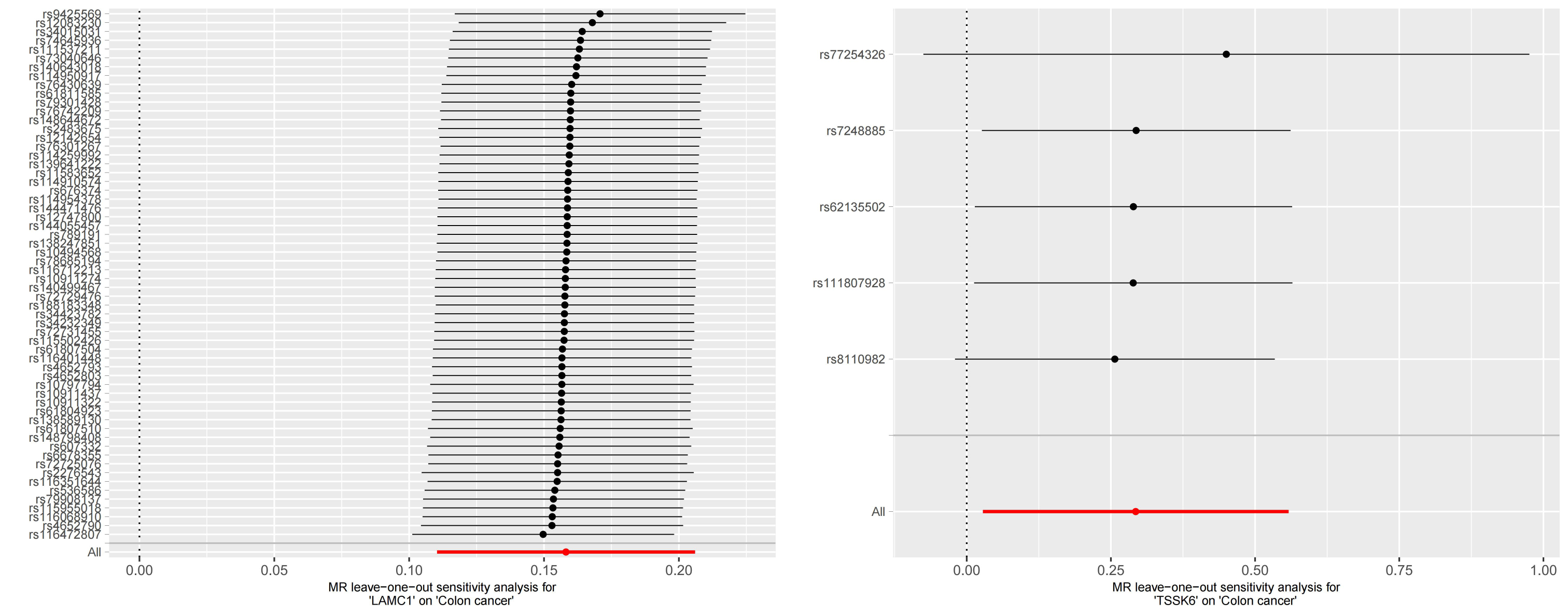


**Fig. S6** Leave-one-out sensitivity analysis for the causal relationship between 2 significant genes and colon cancer.


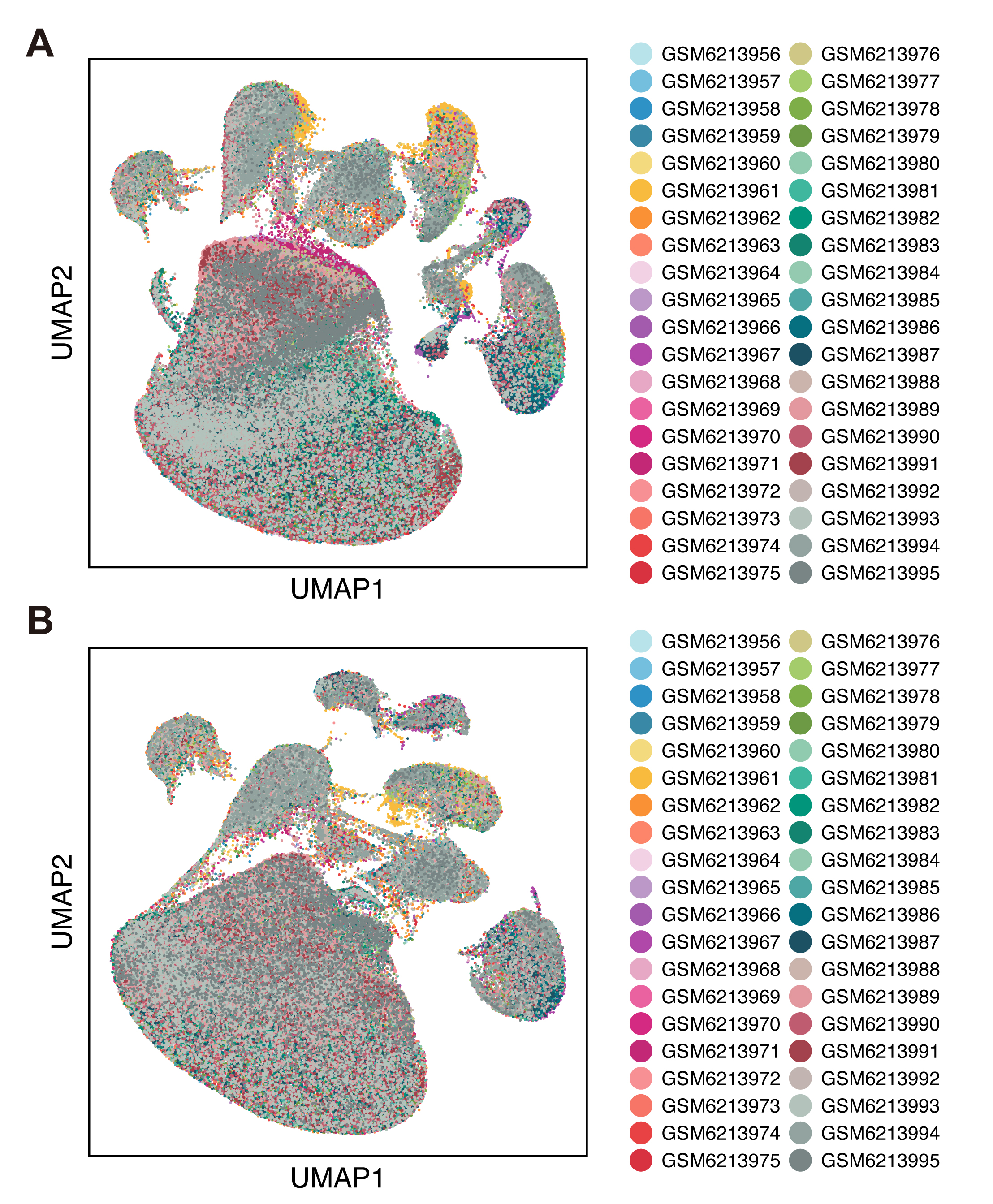


**Fig. S7** UMAP plots comparing the cell clustering before and after batch correction. (A) Cell clustering before batch correction. (B) Cell clustering after batch correction.

**

**

**Fig. S8** Correlation analysis regarding the expression levels of target genes and key T cell exhaustion markers (PD-1, PD-L1, TIM-3) as well as the immune-suppressive cytokine IL-10. (A) TFRC vs. CD274. (B) TFRC vs. CD279. (C) TFRC vs. HAVCR2. (D) TFRC vs. IL-10. (E) TNSF14 vs. CD274. (F) TNFSF14 vs. CD279. (G) TNFSF14 vs. HAVCR2. (H) TNFSF14 vs. IL-10. (I) LAMC1 vs. CD274. (J) LAMC1 vs. CD279. (K) LAMC1 vs. HAVCR2. (L) LAMC1 vs. IL-10. (M) PLK1 vs. CD274. (N) PLK1 vs. CD279. (O) PLK1 vs. HAVCR2. (P) PLK1 vs. IL-10. (Q) TYMS vs. CD274. (R) TYMS vs. CD279. (S) TYMS vs. HAVCR2. (T) TYMS vs. IL-10. (U) TSSK6 vs. CD274. (V) TSSK6 vs. CD279. (W) TSSK6 vs. HAVCR2. (X) TSSK6 vs. IL-10.
